# Recent Advances in Cardiac Surgery

**Published:** 1913-05-10

**Authors:** 


### NEW DEVELOPMENTS IN MEDICINE.

### IV.—Recent Advances in Cardiac Surgery.

Less than fifty years ago a surgeon, who at the time was perhaps the best known exponent of his science and art, pessimistically wrote: "The only organ that supremely baffles our skill is the heart." And yet Billroth was perfectly aware that cardiac surgery was not a novelty and that the heart had never been looked upon as sacrosanct by the surgeons. Himself an able student of the history of medicine, he must have known that Avicenna had reported the remarkable vitality of the heart in a man who recovered after the apex of his heart had been almost sliced away by a spear thrust. Similarly he must have been aware that among his own contemporaries there were men who had bravely attacked the heart or the larger blood vessels in the attempt to relieve conditions which threatened the lives of their patients. But he was also perfectly aware that none of these attempts had succeeded, and that the curious cases recorded in history in regard to the earliest attempts at cardiac surgery were on a par with the daring methods of Susrutha, who sutured the peritoneum by means of ants' heads—brilliant experiments that led to nothing in the way of material advance. For Billroth, as for all his contemporaries, the heart was an organ with *noli me tangere* writ large across it.

### The History of Modern Advance.

Only the advance in aseptic surgery and the refinements of the more modern operative technique have made it possible for surgeons to show that the dictum in regard to the untouchability of the heart no longer holds. In the late 'eighties of the last century an Italian surgeon successfully stitched up a stab wound of the thorax in which the heart was slightly involved his patient recovered and to all intents and purposes was normal and healthy three years later. From that time several cases were recorded of wounds of the heart which had been successfully dealt with by surgical methods. In all the material point was that strict asepsis was practised in the technique and that the actual suturing was done quickly and delicately. But active surgical interference was as yet only discussed; when it was tried it was tried as a last resort, as a sort of forlorn hope which promised little and held out grave dangers of immediate catastrophe. Surgery had advanced so rapidly and its conquests had been so great that these discussions touched on possibilities which ten years before had been undreamed of.

### The Proposed Surgery of Mitral Stenosis.

Thus a Russian surgeon put forward the view that surgical interference held out a chance of relief in cases of extreme stenosis of the mitral orifice; his suggestion was that a narrow sharp pointed tenotome, specially made for the purpose, should be passed into the heart and the orifice of the valve slit; the operation, as outlined, was to be in the nature of a tenotomy for which the word cardiotomy was proposed. A somewhat similar proposal was made by Sir Lauder Brunton some years later, but so far as we are aware the operation has never been performed on the living subject, largely owing to the difficulty, perhaps, of selecting a suitable case. The fact that such an operation has been proposed and has been seriously discussed shows, however, that the advance in cardiac surgery has been singularly rapid and phenomenal during the last two decades.

### The Kiel Pioneers.

This advance is prominently associated with the name of Professor Rehn, who may claim to be one of the foremost pioneers of cardiac surgery. Rehn has operated on many cases with striking success, and some of the cases published by him are wonderful examples of what modern surgery is able to accomplish in the hands of a skilful and daring operator. He has sutured the ventricle and auricle and the great vessels leading out of the heart, and he has proved that it is possible to constrict the heart for several minutes while a ligature is being applied to the edges of an incision without endangering the life of the patient. So far the most common operations have been for the relief of traumatic lesions, such as stabs, bullet wounds, and injuries inflicted by crushing of the ribs. In the case of purely pathological lesions the field is very much more restricted, and the best results have been obtained in cases of lesions of the pericardium. The evacuation of pus from the pericardial sac is now an operation which is included in all the larger manuals of surgery and one with which every hospital surgeon is supposed to be familiar.

### Surgery of the Blood Vessels.

Through the researches of German surgeons it has been shown that a number of methods are available for operating on the large vessels, and this again has extended the field of what may be called the surgery of the vascular system. The pioneer in this line was Matas, the American surgeon, whose operation for aneurysmorraphy, or plication of the walls of an aneurysmal sac, has now been done so often that we are in a position to compare its after results with those of more conservative methods. Undoubtedly the most signal new method in recent times has been Trendelenburg's operation for pulmonary embolus. Surgeons who were present at the Berlin Congress in the spring of 1908 will remember the enthusiasm which greeted the professor's demonstration of his first case—that of a calf which recovered rapidly from this unusually severe operation and lived until it was killed in order to test post-mortem the results of the operation on the heart itself and the great vessels. Since that demonstration the operation has been tried in several cases so far, unfortunately, with indifferent success. The reason for this apparent failure is to be found in the fact that the operation is necessarily performed under extremely trying conditions; it is an operation of emergency in the strictest sense of the term, and there is no time or opportunity for preparation of patient, instruments, or surgeon. In fact the simplicity of the technique is such that the operation can be carried out with a pocket-knife and a hairpin, although the risk of sepsis in such a case is of course enormously increased. In the larger German clinics the operation instruments for the Trendelenburg operation in cases of pulmonary embolism are always kept in the ward ready sterilised so that they are available at a moment's notice. Even with such preparation it is difficult to increase the chances of success, since the onset of the symptoms is, in many cases, so quick that the surgeon must operate at once if he wishes to give the patient that slight chance of recovery which the operation holds out. That it undoubtedly does hold out some chance is certain, for cases have survived the operation, only, unfortunately, to succumb a few days later from the effects of sepsis entailed by the rough-and-ready methods used in the emergency.

### Some Conclusions.

Enough has been said to outline broadly the advance that has been made in cardiac surgery during recent years. In order to practise these modern methods successfully the operator must have at hand modern instruments and modern aids. The problem of instruments does not present much difficulty, since the instrumentarium required is comparatively simple, and only a few new modifications are required. But the aids are another matter. Operations on the thorax are best performed under the almost ideal conditions obtainable in a pressure cabinet of the Brauer or low-pressure type, and to fit a modern operating theatre with such a cabinet entails some expenditure which medical committees may not be inclined to sanction. It is worth while pointing out, however, that no modern hospital can, or ought to, afford to allow itself to be handicapped by the want of proper aids in performing operations or giving treatment along modern lines which have proved to be good lines. As we have already said, in referring to the developments in regard to gastric surgery, the surgeon should insist upon having an outfit which is in accordance with modern requirements. A little less expense upon the operating theatre itself, a little economy in the provision of ultra-refined safeguards against dust, shadows and noise—that ultra-refinement which is responsible for the exaggerated cost of operating theatres in some modern hospitals, and which really does not conduce to a decrease in the mortality commensurate with the great expense—and a little broad-mindedness will enable committees to meet the wants of the surgeon without unduly straining the financial resources of the institution.

Cardiac surgery is no longer in its experimental stage; it has progressed beyond that, and has become a recognised branch of general surgery with which the ordinary hospital surgeon has to be familiar. Unless a hospital can give its patients the benefit of these recent advances in treatment it does not deserve to style itself a modern and up-to-date institution properly equipped. This is the moral of the history of advance along all lines, that it calls for equal advances in administration and equipment, and that it entails greater expense and a higher cost in upkeep. It is useless to grumble or cavil at such increased burdens being thrown upon the institutions. To be modern and efficient a hospital must not grudge expense if that expense is devoted to amplifying and modernising its means of treatment and thereby increasing its scope of usefulness. Our whole object in drawing attention to these recent developments in medicine is to impress upon hospital workers the fact that modern requirements are constantly increasing. Hospital secretaries and others responsible for the administration of our institutions should make this fact a powerful appeal to the generosity and logical sense of their supporters, and by pointing out in what manner the new requirements aid in improving treatment and in raising the percentage of cures and of cases relieved, enable these supporters to keep in mind the broad general outlines of what the equipment of an efficient hospital means in modern times.

